# Zuschauendes Verhalten an Unglücksorten – nicht immer ist es „Schaulust“, nicht immer sind es „Gaffer“

**DOI:** 10.1007/s00103-022-03585-0

**Published:** 2022-09-19

**Authors:** Harald Karutz

**Affiliations:** MSH Medical School Hamburg, University of Applied Sciences and Medical University, Am Kaiserkai 1, 20457 Hamburg, Deutschland

**Keywords:** Wahrnehmung, Einsatzpraxis, Prävention, Sicherheitsbedürfnis, Katastrophentourismus, Awareness, Emergency practice, Prevention, Need for security, Disaster tourism

## Abstract

Fast täglich berichten Medien darüber, dass Rettungsarbeiten an Unglückorten von „Gaffern“ und „Schaulustigen“ behindert werden. Ein derartiges Verhalten wird von Einsatzkräften sowie der breiten Öffentlichkeit kritisiert und als unethisch, verantwortungslos oder auch als Ausdruck gesellschaftlicher Verrohung betrachtet. Emotional ist das Thema stark aufgeladen.

Mit diesem Artikel soll ein Überblick zu den Hypothesen und Theorien gegeben werden, mit denen zuschauendes Verhalten erklärt werden kann. Mittels einer Literaturrecherche wurden biologische bzw. ethologische, individual- und sozialpsychologische Erklärungsansätze für zuschauendes Verhalten an Unglücksorten identifiziert. Diese einzelnen Ansätze wurden in einem integrativen Rahmenmodell zusammengeführt.

Es zeigt sich, dass zuschauendes Verhalten an Unglücksorten keineswegs allein auf „Neugier“ und „Schaulust“ zurückzuführen ist. Vielmehr muss von einem komplexen Zusammenwirken biologischer bzw. ethologischer, individual- und sozialpsychologischer Motive mit gesellschafts-, ereignis- und personenbezogenen Moderatorvariablen ausgegangen werden. Die pauschale Bezeichnung von Zuschauern an Unglücksorten als „Gaffer“ bzw. „Schaulustige“ wird der Komplexität des Phänomens somit nicht gerecht. Zuschauendes Verhalten an Unglücksorten ist individuell unterschiedlich und multifaktoriell begründet. Erst ein dementsprechend umfassendes Verständnis der Problematik bietet eine solide Grundlage für die Ableitung geeigneter Präventions- und Interventionsstrategien.

## Abstract (en)

Almost every day, the media report that emergency services at accident sites are hindered by rubberneckers. Such behaviors are criticized by firefighters, rescue service employees, and the general public and regarded as “unethical,” “irresponsible,” or even as an expression of social brutalization. Emotionally, the topic is highly charged.

This article gives an overview of the hypotheses and theories that can be used to explain watching behavior. A literature search was conducted to identify biological, ethological, individual, and social psychological explanatory approaches for watching behavior at accident sites. These individual approaches are brought together in an integrative framework model.

It turns out that watching behavior at accident sites is by no means solely due to “curiosity” and “desire to look.” Rather, a complex combination of biological, individual, and social psychological motives with social, event, and personal moderator variables must be assumed. The blanket designation of spectators at accident sites as rubbernecks thus does not do justice to the complexity of the phenomenon. Watching behavior at accident sites is individual and multifactorially justified. Only a correspondingly comprehensive understanding of the problem provides a solid basis for the derivation of suitable prevention and intervention strategies.

## Einleitung

Medien berichten häufig darüber, dass „Gaffer“ bzw. „Schaulustige“ an Unglücksorten im Weg gestanden, Einsatzkräfte behindert oder auf unakzeptable Weise Fotos und Videoaufnahmen angefertigt haben. Ein solches Verhalten stößt in der Öffentlichkeit auf Kritik: Den Menschen, die bei einem Notfallgeschehen zuschauen, wird vorgeworfen, aus niedrigen und verabscheuungswürdigen Beweggründen zu handeln.

Auf diese Weise ist zum einen der Eindruck entstanden, zuschauendes Verhalten sei ein ausschließlich negativ einzuschätzendes, moralisch verwerfliches bzw. unethisches Verhalten. Zum anderen sieht es so aus, als habe sich erst in der jüngeren Vergangenheit eine Problematik entwickelt, die es zuvor nicht gegeben hat. Bei einer näheren Betrachtung zeigt sich jedoch, dass zuschauendes Verhalten an Unglücksorten eine keineswegs neue, aber sehr komplexe Problematik darstellt, die „multifaktoriell“ verursacht und von zahlreichen Moderatorvariablen beeinflusst wird. Bloße Forderungen nach härteren Strafen für „Gaffer“ reichen daher auch nicht aus, um diesem Phänomen gerecht zu werden. Effektive Präventions- und Interventionsstrategien müssen vielmehr berücksichtigen, wie unterschiedlich zuschauendes Verhalten an Unglücksorten motiviert sein kann. Hierzu geben die folgenden Ausführungen einen orientierenden Überblick^1^. Zugleich wird auf bestehende Forschungsdefizite hingewiesen. Grundlage der Ausführungen ist eine Literaturrecherche, die im Januar 2022 in der Fachdatenbank des Bundesamtes für Bevölkerungsschutz und Katastrophenhilfe^2^ begonnen und im weiteren Verlauf nach dem Schneeballprinzip fortgeführt worden ist.

## Historische Aspekte und die aktuelle Problematik

„Gaffer“ und „Schaulustige“ hat es in der gesamten Menschheitsgeschichte immer schon gegeben. Archaische Rituale, Gladiatorenkämpfe in der Antike sowie öffentliche Folterungen und Hinrichtungen im Mittelalter können hier beispielhaft angeführt werden [3]. Solche Ereignisse waren in früheren Zeiten beliebte Attraktionen: Je mehr Blut geflossen ist und je mehr Schmerzensschreie zu hören waren, umso „besser“ war es aus Sicht des Publikums [4].

In vielen Publikationen wird aus unterschiedlichen Zeiten und im Kontext unterschiedlicher Situationen über zuschauendes Verhalten berichtet (z. B. [5, 6]). Unter anderem erschien im *Spiegel* bereits vor über 20 Jahren ein Beitrag, in dem „Schaulust“ als ein „neues“ Phänomen beschrieben wurde, das bei Einsatzkräften große Besorgnis und Verärgerung auslöst [7]. Etwa zur gleichen Zeit wiesen Studien der Bundesanstalt für Straßenwesen darauf hin, dass an Unfallorten – abhängig von der Tageszeit, dem Ereignisort und weiteren Variablen (Wetterbedingungen, Art des Unglücks usw.) – meist zwischen 16 und 26 zuschauende Personen anwesend seien [8, 9].

Verlässliche Zahlen zur Anwesenheit von Zuschauern in *aktuellen* Notfallsituationen gibt es zwar nicht: Der öffentlichen Empörung über Zuschauer an Unglücksorten steht das weitgehende Fehlen einer entsprechenden Empirie gegenüber. Eine derart aktuelle Brisanz der Thematik lässt sich so, wie sie in manchen Medienberichten beschrieben wird [10], tatsächlich jedoch *nicht* erkennen.

Dass die Anwesenheit von Zuschauern an Unglücksorten aus verschiedenen Gründen problematisch sein kann, ist unstrittig [11–13]:

- Für Notfallpatienten, Angehörige und weitere Ereignisbeteiligte (z. B. Unfallverursacher) stellen Zuschauer einen zusätzlichen Belastungsfaktor dar; Schamgefühle werden verletzt.
- Die Hilfeleistung kann beeinträchtigt werden, weil Zuschauer im Weg stehen bzw. Rettungsmaßnahmen behindern.
- Foto- und Videoaufnahmen verstoßen gegen Persönlichkeitsrechte.
- Einsatzkräfte fühlen sich durch Zuschauer gestört und einem besonderen Erfolgs- oder Handlungsdruck ausgesetzt.
- Herabwürdigende, kritische Kommentare von Zuschauern werden von Einsatzkräften als demotivierend erlebt.

Zuschauer können an Unglücksorten auch zusätzliche Sachschäden verursachen oder Spuren verwischen, die zur Aufklärung des Ereignisses hätten beitragen können. Nicht zuletzt setzen sie sich einer psychischen und physischen Eigengefährdung aus. Dennoch ist nicht auszuschließen, dass ein eigentlich uraltes und urmenschliches Phänomen derzeit (v. a. medial) überbewertet und etwas einseitig betrachtet wird.

## Erklärungsansätze

Forschung zum zuschauenden Verhalten an Unglücksorten ist mit diversen methodischen Schwierigkeiten verbunden: Befragungen unmittelbar in einer Notfallsituation lassen sich z. B. kaum realisieren. Retrospektive Interviews dürften in hohem Maße Verzerrungseffekte sowie sozial erwünschte Aussagen beinhalten und Medienberichte über das Verhalten von Zuschauern stellen erst recht keine verlässlichen Quellen dar.

Dennoch liegen unterschiedlichste Hypothesen und Theorien vor, die bislang allerdings weitgehend unverbunden nebeneinanderstehen. Sie sind sicherlich nicht alle in gleichem Maße relevant; sie werden auch nicht bei jedem Individuum und in jeder (Notfall‑)Situation eine Rolle spielen. Aber sie zeigen eben die Vielfalt *möglicher* Erklärungsansätze auf, die bei einer unvoreingenommenen Analyse von zuschauendem Verhalten zumindest *in Betracht zu ziehen* sind. Im Folgenden werden die verschiedenen Erklärungsansätze für zuschauendes Verhalten am Unglücksort beschrieben. Sie lassen sich in 3 Gruppen einteilen: biologische bzw. ethologische, individual- und sozialpsychologische Motive.

### Biologische bzw. ethologische Motive

Experimentelle Untersuchungen haben gezeigt, dass Menschen, die von jeglichen Umgebungsreizen abgeschottet werden, bereits nach relativ kurzer Zeit psychische Störungen entwickeln („sensorische Deprivation“; [14]). Eine vielleicht triviale Erkenntnis lautet daher: Ein Mensch schaut zu, weil er es *kann* und weil er so *angelegt* ist, dass er seine Sinnesorgane nutzt [3].

In außergewöhnlichen Situationen, mit denen Menschen überraschend oder unvorbereitet konfrontiert werden, ist regelmäßig auch eine *reflexhafte Orientierungsreaktion* zu beobachten. Eine solche, instinktiv verankerte Reaktion ist sinnvoll, weil auf diese Weise potenzielle Gefahren rasch erkannt werden können. Die oftmals kritisierte Staubildung nach Unfällen im Straßenverkehr kann auf diese Weise verständlich gemacht werden: So ist es richtig abzubremsen, wenn man als Autofahrer eine nicht eindeutig identifizierbare Situation wahrgenommen hat: Beispielsweise könnten Trümmerteile auf der eigenen Fahrbahn liegen oder es könnte ein Ausweichmanöver erforderlich sein [3].

Möglicherweise resultiert zuschauendes Verhalten aber auch aus einer mehr oder weniger unbewussten *Sorge um die eigene Art*: Wenn das Leben eines anderen Menschen gefährdet ist, wird von Artverwandten u. U. Anteil genommen, und zwar auch dadurch, dass man räumliche Nähe zum Betroffenen sucht: „Das hat nichts mit Voyeurismus zu tun – im Gegenteil. … Das Leid anderer lässt uns nicht kalt, wir sind berührt und besorgt, und gerade, dass wir nicht helfen können, lässt uns nicht kalt. Das führt zwangsläufig dazu, dass wir immer wieder hinüberschauen, und eben nicht einfach weiterfahren. … Das macht uns als Menschen aus“ [15].

Zurückgeführt wird ein solches Verhalten auf Relikte aus der Entwicklungsgeschichte des Menschen. So wird es vor Tausenden Jahren unmittelbar lebensnotwendig gewesen sein, sich in bedrohlichen Situationen zu unterstützen oder sich z. B. bei einem Tierangriff gemeinsam zu verteidigen. Ein solches Verhaltensmuster könnte noch immer dazu führen, dass eine instinktive Hinwendung zu einem Notfallgeschehen erfolgt [15] – auch wenn dies heute nicht mehr uneingeschränkt sinnvoll ist und eher dazu führt, im Weg zu stehen bzw. Rettungsarbeiten zu beeinträchtigen.

### Individualpsychologische Motive

Das wohl naheliegendste Motiv für zuschauendes Verhalten – *Neugier* – ist mittlerweile oftmals negativ konnotiert („Sei nicht so neugierig!“). Gleichwohl ist ohne menschlichen Erkenntnisdrang und die beständige Suche nach neuen Informationen keine persönliche Weiterentwicklung möglich [16]. Grundsätzlich ist es also ebenfalls nicht zu verurteilen, wenn jemand sich dafür interessiert, was an einem Unglücksort geschehen ist. Es sollte auch nicht immer nur davon ausgegangen werden, dass Zuschauer unbedingt Verletzte oder Verstorbene sehen möchten: Ein „interessiertes Hinschauen“ kann sich ebenso darauf beziehen, einen Unfallhergang nachzuvollziehen, zu erkunden, wie es den Beteiligten geht oder wie Rettungsmaßnahmen durchgeführt werden.

Mit einem „interessierten Hinschauen“ im direkten Zusammenhang steht u. U. auch der Wunsch, aus dem Wahrgenommenen etwas *lernen* zu können: Eventuell fragt ein Zuschauer sich, was er selbst tun könnte, um ein solches Unglück zu verhindern, oder wie er sich selbst verhalten sollte, wenn ihm einmal etwas Ähnliches passiert [17]. Notfälle lassen es zu, sich mit der Möglichkeit einer eigenen existentiellen Bedrohung zu beschäftigen, ohne sich dabei selbst in Gefahr begeben zu müssen; sie erlauben es, vergleichbare Szenarien „mental durchzuspielen und innere Haltungen dazu zu erproben“ [18]. Auf diese Weise sind Notfälle „Lern- und Übungsgelegenheiten“ und sie enthalten auch ein *Bildungspotenzial* [11].

Die Befriedigung eines *Sicherheitsbedürfnisses* könnte ebenfalls eine Rolle spielen: So erhöht die Wahrnehmung eines verletzten anderen das Bewusstsein, selbst eben *nicht* verletzt zu sein [18]. Zudem könnte der Anblick eines verletzten, also „geschwächten“ oder „gescheiterten“ anderen einem selbst ein Gefühl von Macht und Stärke verleihen – was wiederum das eigene Sicherheitsempfinden intensiviert [1, 3].

Unbestritten ist außerdem, dass Notfallgeschehen Dramatik, „Nervenkitzel“ und „Action“ bieten, wie man es sonst womöglich nur aus Kinofilmen oder dem Fernsehen kennt. Anspannung wird dabei angenehm, d. h. als *Lustgewinn* erlebt. Das Konzept des „sensation seeking“ (Sensationssuche) bezieht sich genau darauf: Demnach ist jeder Mensch bestrebt, stets den für ihn angenehmsten und damit wünschenswerten Erregungslevel zu erreichen [19]. Bei einem Notfall zuzuschauen, kann einen Beitrag dazu leisten. Nicht ausgeschlossen ist, dass auch ein tatsächliches *Ergötzen*, eine wirkliche *Freude* am Leiden anderer sowie ein *sexueller Lustgewinn* von Bedeutung sind – dies dürften aber Ausnahmefälle sein [7].

Zahlreiche Zuschauer wird das, was sie an einem Unglücksort zu sehen bekommen, eher betroffen machen oder verängstigen. Für diesen Personenkreis kann es hilfreich sein, die Arbeit der Rettungskräfte zu verfolgen, weil dadurch „das beruhigende Gefühl vermittelt [wird], dass man sich in einem Notfall auf solche Helfer verlassen kann“ [11]. Zudem kann die *Bewältigung einer Notfallerfahrung* im weiteren Verlauf begünstigt werden, wenn diese nicht nur mit Ohnmachts- und Hilflosigkeitsgefühlen, sondern ebenso mit dem Anblick von Hilfeleistungen verknüpft ist [20]. 75 % der Kinder, die zu ihrem Erleben unterschiedlicher Notfallsituationen befragt worden sind, beschrieben es beispielsweise als etwas Positives, dass sie laufende Rettungsmaßnahmen beobachten konnten [20].

Starke psychische Betroffenheit kann auch dazu führen, dass zuschauende Personen sich – „starr vor Schreck“ – nicht mehr von einem Unglücksort abwenden können: Entsprechende *Denk- und Handlungsblockaden* bei Zuschauern sind empirisch belegt; zuschauendes Verhalten in Notfallsituationen könnte insofern als Symptom einer psychischen Überforderung verstanden werden [21].

Der „Zeigarnik-Effekt“ beschreibt schließlich, dass unterbrochene, nicht zu Ende geführte oder verfolgte Handlungen oftmals als besonders belastend erlebt werden [22]. Dies lässt sich auf Notfallsituationen übertragen: Ein „psychologischer Abschluss“ kann nicht gefunden werden, solange Rettungsarbeiten fortgeführt werden und unklar ist, wie das Geschehen ausgeht. Also bleibt Zuschauern sozusagen kaum etwas anderes übrig, als stehen zu bleiben und weiter zuzuschauen – beispielsweise bis zum Abtransport eines Verletzten oder bis zu den letzten Aufräumarbeiten an einer Einsatzstelle; ansonsten bleibt das Wahrgenommene jedenfalls „offen“ und unabgeschlossen.

### Sozialpsychologische Motive

Über die bereits genannten individualpsychologischen Erklärungsansätze hinaus kommen weitere aus dem Bereich der Sozialpsychologie hinzu, die sich aus der Interaktion mit anderen Menschen ergeben. Jeder Mensch orientiert sich z. B. mehr oder weniger stark am Verhalten seiner Mitmenschen. Dahinter steht die Vermutung, dass es gewiss einen guten Grund geben wird, sich so zu verhalten, wie andere dies tun [23]. Unter Umständen kommt es auf diese Weise allerdings zu einer „kollektiven Fehlinterpretation“, d. h., die Lage wird so eingeschätzt, als ob man zuschauen müsse – obwohl es bei einem Notfallgeschehen objektiv betrachtet viel sinnvoller wäre, rasch weiterzugehen (oder natürlich Hilfe zu leisten; [11]).

In engem Zusammenhang mit der *Orientierung an anderen Menschen* kann auch auf *pluralistische Ignoranz* hingewiesen werden. Demnach wird ein eventuell vorhandenes schlechtes Gewissen beim Zuschauen dadurch vermindert, dass jeder einzelne wiederum andere beobachtet, die sich ja ebenso verhalten wie man selbst. Daraus ergibt sich die Legitimation: „Wenn alle zuschauen, darf ich das auch!“ [23].

Wenn Menschen ein ausgeprägtes Bedürfnis haben, sich anderen *zugehörig* zu fühlen, werden sie sich einer Gruppe von zuschauenden Personen außerdem eher anschließen, als sich von ihr zu distanzieren. Gegebenenfalls bleibt man stehen und schaut ebenfalls zu: „Ich kann doch nicht als einziger weg gehen, wenn alle anderen stehen bleiben und zuschauen“ [24].

Möglicherweise entsteht durch das Zuschauen an einem Unglücksort sogar erst die Gemeinschaft, die „eine spezifische, *soziale Gegenwelt* zu einem besonders grausamen Geschehen konstituiert. Gerade dieses ‚Kollektiv‘ entsteht und verbündet sich dann, wenn es darum geht, etwas Schrecklichem etwas Positives entgegen setzen zu können“ [11]: Demnach würde sich, so die Annahme, „eine Gemeinschaft der Gaffer in schützender und distanzierender Weise vor das Schicksal [der Verunglückten schieben]“ [18].

*Positive Verstärkung* bzw. *Belohnung* ist für das zuschauende Verhalten an Unglücksorten sicherlich ebenfalls von Bedeutung [13]: „Wer von einem Unglück berichten kann, wird durch interessierte Aufmerksamkeit seiner späteren Zuhörer ‚belohnt‘. Kann man nicht nur in Worten schildern, sondern auch noch (Foto- und Video-) ‚Beweise‘ vorlegen, wird die Anerkennung im eigenen Publikum u. U. sogar noch größer sein“ [11]. In diesem Zusammenhang ist außerdem darauf hinzuweisen, dass nicht wenige Nachrichtenredaktionen Honorare für Fotos und Videos von „Leserreportern“ zahlen. Nicht unerwähnt bleiben soll auch, „dass es mitunter die gleichen Medien sind, die zu Leserreportagen aufrufen, wie die, in denen dann empört über Zuschauer an Unglücksorten berichtet wird“ [11].

Speziell für das Anfertigen von Fotos und Videos an Unglücksorten können noch zahlreiche weitere mögliche Motive angeführt werden, was jedoch den Rahmen dieser Darstellung sprengen würde. Neben dem Wunsch nach Anerkennung und Aufmerksamkeit könnte hier u. a. der Rückgriff auf vertraute Handlungsroutinen bei gleichzeitig fehlendem ethischen Problembewusstsein von Bedeutung sein. Es ist aber auch nicht ausgeschlossen, dass die Nutzung einer Kamera bzw. eines Smartphones einen Distanzierungsversuch darstellt, indem gerade das technische Medium für einen etwas größeren (psychischen) Abstand vom Geschehen sorgt – wie es in ähnlicher Weise z. B. auch von Journalisten in Kriegsgebieten berichtet wird [11]. Diese Überlegungen müssen in weiterführenden Studien jedoch erst noch genauer betrachtet werden.

### Moderatorvariablen

Neben den dargestellten biologischen bzw. ethologischen Erklärungsansätzen sowie individual- und sozialpsychologischen Überlegungen sind verschiedene *Moderatorvariablen* zu berücksichtigen, die zuschauendes Verhalten entweder fördern oder hemmen ([11]; Tab. 1):

- *Ereignisbezogene Variablen* erklären, warum bestimmte Unglücke besonders viele Zuschauer „anziehen“, während andere offenbar weniger interessant sind. Ein plötzlich auftretendes Schadensereignis mit großen Zerstörungen wirkt z. B. „attraktiver“ als ein sich langsam anbahnendes, das vergleichsweise geringe Schäden anrichtet [3, 13].
- *Gesellschaftsbezogene Variablen* resultieren aus der übergeordneten gesellschaftlichen Situation. Hier sind insbesondere die geltenden Normen und Werte relevant (was ist erwünscht, was nicht; was wird legitimiert, was nicht), aber beispielsweise auch die Frage, wie oft im eigenen Umfeld Notfälle überhaupt erfahrbar sind. Je seltener dies der Fall ist und je unbekannter bestimmte Ereignisse sind, umso mehr wird es interessant und lohnend erscheinen, sich an einem Unglücksort umzuschauen [3, 13].
- *Personenbezogene Variablen* ergeben sich schließlich aus der Person sowie dem unmittelbaren Umfeld einer bzw. eines (potenziell) Zuschauenden [3, 13]. Vorerfahrungen, in der Erziehung Vermitteltes, Empathie- und Selbsthilfefähigkeit, aber auch die Reaktion von Angehörigen, Freunden und Bekannten, die aktuelle Stressbelastung sowie Aspekte der beruflichen Sozialisation („Berufsethos“) sind hier beispielhaft zu nennen [25].

**Table 1:** 

Variable	Fördernde Wirkung	Hemmende Wirkung
*Ereignisbezogen*		
Häufigkeit	Selten	Oft
Eintritt	Plötzlich, abrupt	Sich langsam anbahnend
Kräftewirkung	Stark	Schwach
Schadensausmaß	Hoch	Gering
Schaden	Menschen	Sachwerte
Prominenz Geschädigter	Hoch	Gering
Emotionale Aufladung	Hoch	Gering
*Gesellschaftsbezogen*		
Erfahrungspotenzial	Hoch	Gering
Sicherheitslevel	Hoch	Gering
Normen und Werte	Unklar oder widersprüchlich	Klar und konsistent
Ausmaß ethischer Reflexion	Gering	Hoch
Medienberichterstattung	Wirkung kann sowohl fördernd als auch hemmend sein	
*Personenbezogen*		
Vorerfahrungen	Positiv	Negativ
Erziehungseinfluss	Je nach Gestaltung unterschiedlich	
Empathiefähigkeit	Hoch	Gering
Angehörige, Freunde und Bekannte	Belohnung für zuschauendes Verhalten	Sanktionierung von zuschauendem Verhalten
Hilfefähigkeit	Hoch	Gering
Stressbelastung	Hoch	Gering
Berufliche Sozialisation	Je nach Berufsgruppe unterschiedlich	
Geschlecht	Wirkung kann sowohl fördernd als auch hemmend sein, abhängig vom Zusammenwirken mit anderen Moderatorvariablen	

### Ableitung eines Rahmenmodells

**Figure 1:**
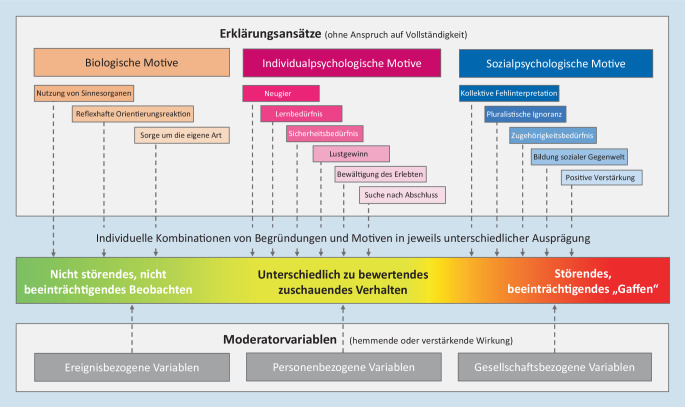

Ein integratives Rahmenmodell, das sämtliche relevanten Wirkmechanismen zusammenfasst, zeigt Abb. 1. Sowohl die einzelnen Erklärungsansätze aus den unterschiedlichen wissenschaftlichen (Teil‑)Disziplinen als auch die ereignis-, personen- und gesellschaftsbezogenen Moderatorvariablen sind hier in einem Zusammenhang zu betrachten: Aus ihrem interdependenten Zusammenspiel können sich in jedem Einzelfall, d. h. bei jeder einzelnen *Person* und in jeder einzelnen *Situation*, völlig unterschiedliche Arten und Ausprägungen zuschauenden Verhaltens ergeben. Zuschauer können z. B. Betroffene, Anteilnehmende, Neugierige, Interessierte, Schockierte, Besorgte, Schutzbedürftige, Gemeinschaft- oder Einen-„Kick“-Suchende, Nach-Aufmerksamkeit-Strebende oder Verunsicherte sein. Entsprechend unterschiedlich ist das jeweilige Verhalten auch zu bewerten.

Der verallgemeinernde Begriff *Gaffer* sollte daher nach Möglichkeit nicht verwendet werden, weil er ausschließlich negativ konnotiert ist und auf einer letztlich unbelegten Annahme basiert [26]. Gleiches gilt für den verallgemeinernden, stigmatisierenden Begriff „Schaulust“, weil er zuschauenden Personen pauschal unterstellt, dass es ihnen vorrangig um einen „Lustgewinn“ gehen würde. Die vielen anderen, für die Entstehung von zuschauendem Verhalten ebenfalls relevanten Einflussfaktoren bleiben bei einer Verwendung dieser Begriffe unberücksichtigt.

Vor diesem Hintergrund wird angeregt, in der rettungsdienstlichen Einsatzpraxis nur noch zwischen 2 *beschreibenden* und weniger interpretierenden Kategorien zu differenzieren:

1. Zuschauer bzw. „Beobachter“ sowie
2. Störer bzw. Gefährder.

In einer Notfallsituation sollte auf eine Analyse der unterschiedlichen, zumindest anteilig vorhandenen Motive bewusst verzichtet werden, auch weil sie unmittelbar im Geschehen ohnehin kaum leistbar ist [11]. Stattdessen sollte allein die Frage nach der einsatztaktischen Relevanz in den Fokus gerückt werden: Um „Störer“ bzw. „Gefährder“ handelt es sich demnach, wenn negative Effekte für die Betroffenen, die Einsatzkräfte oder die laufenden Rettungsarbeiten verursacht werden. Zuschauer, die aus größerer Distanz zuschauen, ohne in irgendeiner Weise für eine Behinderung der Rettungsarbeiten zu sorgen, sind damit ausdrücklich nicht gemeint. Hier sollte auch kein Feindbild aufgebaut werden, wenn es dafür überhaupt keine Veranlassung gibt [1].

Die Gruppe der tatsächlichen Störer bzw. Gefährder dürfte relativ klein sein. In Schätzungen wird davon ausgegangen, dass sie nur rund 20 % der insgesamt bei Unglücken zuschauenden Menschen ausmacht [12]. Dies wiederum bedeutet, dass man der weit überwiegenden Mehrheit der Zuschauer nicht gerecht wird, wenn man ihr grundsätzlich problematisches, störendes Verhalten vorwirft.

## Ableitung von Konsequenzen für Einsatzpraxis und Prävention

Vor dem Hintergrund der bisherigen Überlegungen werden im folgenden Teil des Beitrags noch einige Konsequenzen für die Einsatzpraxis und für die Entwicklung genereller Präventionsstrategien abgeleitet. Auch bei diesen Vorschlägen ist darauf hinzuweisen, dass sie sich zwar theoretisch gut begründen lassen, ihre Wirksamkeit bislang jedoch nicht empirisch überprüft worden ist.

### Hinweise für den Umgang mit Zuschauern

#### Zuschauer akzeptieren und Ruhe bewahren.

Wichtig scheint zunächst einmal, gelassen zu bleiben, entsprechende Situationen zu „ent-emotionalisieren“ und v. a. Ruhe zu bewahren. Sofern Zuschauer nicht im Weg stehen, einen Einsatz behindern und sie auch keine Persönlichkeitsrechte von Betroffenen verletzen, sollte ihr „Gegebensein“ hingenommen und ausgehalten werden [21]. In rund 90 % der Einsätze dürfte dies die effektivste Strategie sein: Für zuschauendes Verhalten an Unglückorten sollte man grundsätzlich *Verständnis* haben – auch wenn es mitunter schwerfallen mag.

Manche, v. a. in den sozialen Medien geäußerten Reaktionen auf zuschauendes Verhalten – beispielsweise die Forderung von drakonischen Sanktionen (bis hin zu langjährigen Haftstrafen sowie zur Amputation von Körperteilen!) – sollten in diesem Zusammenhang größere Besorgnis auslösen als das zuschauende Verhalten selbst. Die Frage, worauf die teilweise sehr emotionalen Reaktionen auf Zuschauer an Unglücksorten eigentlich zurückzuführen sind, verdient eine eigene Betrachtung, die an dieser Stelle jedoch nicht geleistet werden kann. Das Spektrum möglicher Erklärungsansätze reicht hier von der Empörung über die Verletzung persönlicher Moralvorstellungen über wenig differenzierte Prozesse der Selbst- und Fremdwahrnehmung bis hin zu Bedrohungsgefühlen, weil Zuschauer Alleinstellungsmerkmale bzw. auch die sehr besondere Rolle von Einsatzkräften gefährden könnten [27].

#### Einbeziehen.

Denkbar ist, Zuschauer in eine Hilfeleistung einzubeziehen. Am aussichtsreichsten dürfte es dabei sein, einzelne Personen gezielt, freundlich-bestimmt anzusprechen und ihnen eine konkrete, einfach zu erledigende Aufgabe zu erteilen [3, 13]. Insbesondere in größeren Schadenslagen stellen Umstehende ein wertvolles Potenzial dar, das genutzt werden sollte. Die Motivation mitzuhelfen ist – entgegen weitverbreiteter (Fehl‑)Annahmen – oftmals sehr wohl vorhanden [28].

#### Zuschauen verhindern.

Zum Schutz von Betroffenen ist es zweifellos empfehlenswert, den Blick auf sie zu verhindern. Zu diesem Zweck können Decken hochgehalten oder auch spezielle Sichtschutzwände aufgestellt werden [3, 13]. Ein Nachteil dieser Vorgehensweise ist, dass sie zeitaufwändig ist und viele Kräfte bindet, die dadurch nicht mehr für andere Aufgaben zur Verfügung stehen. Zudem muss betont werden, dass die Verhinderung des Zuschauens keine rettungsdienstliche, sondern eine polizeiliche Maßnahme darstellt [29].

#### Auf Störungen reagieren.

Sofern Zuschauer tatsächlich stören – d. h. Rettungsarbeiten behindern oder für die Betroffenen eine Belastung darstellen – sollten sie mit einer freundlich-bestimmten Anweisung aufgefordert werden, zurückzutreten und Platz zu machen. Hilfreich ist es, wenn man solch eine Anweisung inhaltlich begründen kann [3, 13]. Foto- und Handyaufnahmen von Betroffenen sollten zum Schutz von Persönlichkeitsrechten unterbunden werden – auch dies ist jedoch Aufgabe der Polizei und nicht der Rettungskräfte [29]. Ein „Nassspritzen“ o. Ä. von zuschauenden Personen z. B. durch die Feuerwehr ist ebenfalls nicht zu empfehlen, weil es grundsätzlich unverhältnismäßig erscheint und u. U. rechtliche Sanktionen gegen denjenigen nach sich ziehen kann, der auf diese Weise interveniert hat.

#### Paradoxe Interventionen.

Eine weitere grundsätzlich mögliche Handlungsstrategie besteht in einer Art „paradoxer Intervention“, bei der ein Zuschauen ausdrücklich erlaubt werden würde. Medien berichteten in diesem Zusammenhang, wie ein Polizeibeamter nach Unfällen auf der Autobahn „Gaffer“ explizit dazu aufgefordert hat, sich einen Verstorbenen näher anzuschauen [30]. Ob sich daraus eine verallgemeinerbare Empfehlung ableiten lässt, sei dahingestellt.

Tatsächlich wurde vor einigen Jahren auch die Idee diskutiert, nach größeren Schadenslagen bestimmte Bereiche am Rand eines Unglücksortes gezielt für Zuschauer freizuhalten und ggf. sogar Leinwände aufzubauen, um Bilder von der Einsatzstelle dorthin zu übertragen. Der Gedanke war, dass Neugierige ohnehin kommen würden, man sie auf diese Weise aber eher lenken und Beeinträchtigungen der Rettungsarbeiten durch sie auch eher verhindern könnte [31].

#### Regeln für das Zuschauen aufstellen.

Ein ähnlicher Ansatz zielt darauf ab, zuschauendes Verhalten als solches nicht zu verbieten, sondern vielmehr „in geordnete Bahnen zu lenken“. So könnte in den Medien und auch an Einsatzstellen vermittelt werden: „Prinzipiell *darf* zugeschaut werden – aber dies nur dann, wenn bestimmte Regeln eingehalten werden“ [11]:

- Weder eine Person noch ein am Unglücksort abgestelltes Fahrzeug dürfen Einsatzkräfte behindern.
- Mindestabstände von (beispielsweise) 10 Metern werden eingehalten.
- Absperrungen dürfen nicht überschritten werden.
- Anweisungen von Einsatzkräften ist umgehend Folge zu leisten.
- Es dürfen keine Videos und Fotos angefertigt werden, auf denen Betroffene zu sehen sind.

#### Hilfe anbieten.

Wenn es tatsächlich so ist, dass Zuschauer von ihren Wahrnehmungen am Unglücksort *betroffen* sind, sollte konsequenterweise auch darüber nachgedacht werden, ob sie als potenzielle Zielgruppe für *psychosoziale Unterstützungsangebote* betrachtet werden müssten [32]. Dieser Gedanke mag irritieren, weil die öffentliche Diskussion zum Thema eher auf *Bestrafungen* als auf Hilfsangebote fokussiert. Abwegig scheint auch diese Überlegung dennoch nicht.

### Langfristig wünschenswerte Präventionsstrategien

Über kurzfristige Interventionen an Einsatzstellen hinaus scheint es wünschenswert, dass die Problematik von zuschauendem Verhalten an Unglücksorten in Fahrschulen, im allgemeinbildenden Schulunterricht oder im Rahmen der Öffentlichkeitsarbeit von Einsatzorganisationen verstärkt aufgegriffen wird. Hier könnte dafür sensibilisiert und aufgeklärt werden, wann und warum das Zuschauen tatsächlich negative Auswirkungen haben kann. Entscheidend ist aber, dass dies sachlich und nicht mit moralisch erhobenem Zeigefinger geschieht, um beispielsweise kein Reaktanzverhalten zu provozieren. Lediglich holzschnittartig ein „schlechtes Gewissen“ zu verursachen, sollte nicht im Vordergrund stehen. Vielmehr müsste es darum gehen, die Entwicklung von ethischer Reflexionsfähigkeit und Medienkompetenz zu unterstützen.

In diesem Zusammenhang sollten Medien sowie Pressesprecher von Einsatzorganisationen auch nicht nur über eskalierte Konflikte mit aggressiven Störern an Unglücksorten berichten, sondern auch auf hilfreiche und erfreuliche Verhaltensweisen von Menschen fokussieren, die es zweifellos ebenso gibt. In diesem Zusammenhang soll auch nicht verschwiegen werden, dass selbst das zuschauende Verhalten an Unglücksorten durchaus positive Auswirkungen nach sich ziehen kann: Zuschauer tragen u. U. zur Aufklärung eines Unfallhergangs oder einer Straftat bei und sogar Foto- sowie Videoaufnahmen werden gelegentlich dankbar von den Ermittlungsbehörden entgegengenommen [33].

Ein interessantes Projekt hat derzeit die Johanniter-Unfall-Hilfe e. V. initiiert. Rettungswagen werden dabei mit einem QR-Code beklebt, der zuschauende Personen über eine Handynachricht unmittelbar auf problematische Aspekte ihres Verhaltens hinweisen und zum Nachdenken anregen soll [34].

Abschließend scheint erwähnenswert, dass v. a. Lehr- und Führungskräfte in Einsatzorganisationen über die Komplexität der Thematik informiert sind und der unreflektierten Weitergabe stark vereinfachter Narrative entgegenwirken. Außerdem gilt es zu betonen, dass härtere juristische Sanktionen allein keine Lösung der Problematik darstellen, weil sie die Komplexität des Phänomens nicht berücksichtigen [11]. Was bislang bedauerlicherweise fehlt, ist ein umfassendes Präventions- und Interventionskonzept, das mehrere Handlungsansätze und -ebenen miteinander verknüpft; hier besteht noch Entwicklungspotenzial.

## Fazit

Zuschauendes Verhalten an Unglücksorten sollte – wie es bereits vor vielen Jahren gefordert worden ist – „entdämonisiert“ werden [1, 21]. Der Eindruck, dass dieses Phänomen aktuell besonders häufig und in zugespitzter Ausprägung auftritt, dürfte vor allem aus einer einseitig erscheinenden Berichterstattung in den Medien entstehen. Tatsächlich liegen unterschiedlichste Erklärungsansätze für das zuschauende Verhalten vor. Neben „Neugier“ und „Schaulust“ kommen auch Anteilnahme, Betroffenheit und vieles andere mehr infrage.

Weitere Forschung sollte sich darauf konzentrieren, die Motivlage für zuschauendes Verhalten *empirisch* und v. a. *quantifizierbar* aufzuhellen. Zudem sollte untersucht werden, wie sich eine differenzierte Betrachtung dieses Phänomens im Rettungswesen vermitteln lässt, wie der Entstehung von „Feindbildern“ („böse Gaffer vs. gute Helfer“) entgegengewirkt werden kann und wie sich ein konstruktiver, deeskalativer Umgang mit zuschauenden Personen trainieren lässt.

## Footnotes

1 Ein Anspruch auf Vollständigkeit kann nicht erhoben werden. Für eine weitere Vertiefung der Thematik sei ggf. auf weitaus umfangreichere Publikationen verwiesen (z. B. [1, 2]).

2 https://fis.bbk.bund.de/aDISWeb/app?service=direct/0/Home/$DirectLink&sp=SOPAC; Suchbegriffe „Zuschauer“, „Schaulust“ und „Gaffer“. Zugegriffen am 01.03.2022.
